# Performance of Mini Parasep^®^ SF stool concentrator kit, Kato-Katz, and formalin-ethyl acetate concentration methods for diagnosis of opisthorchiasis in Northeast Thailand

**DOI:** 10.1186/s13071-022-05338-z

**Published:** 2022-06-27

**Authors:** Kulthida Y. Kopolrat, Seri Singthong, Narong Khuntikeo, Watcharin Loilome, Chanika Worasith, Chutima Homwong, Chompunoot Wangboon, Patiwat Yasaka, Chatanun Eamudomkarn, Opal Pitaksakulrat, Krisnakorn Tonkhamhak, Arunee Paeyo, Thomas Crellen, Jiraporn Sithithaworn, Paiboon Sithithaworn

**Affiliations:** 1 Faculty of Public Health, Kasetsart University Chalermphrakiat Sakon Nakhon Province Campus, Sakon Nakhon, Thailand; 2 Cholangiocarcinoma Research Institute and Department of Parasitology, Faculty of Medicine, Khon Kaen University, Khon Kaen, Thailand; 3 The Office of Disease Prevention and Control 7 Khon Kaen, Department of Disease Control, Ministry of Public Health, Khon Kaen, Thailand; 4 Department of Surgery, Faculty of Medicine, Khon Kaen University, Khon Kaen, Thailand; 5 Department of Biochemistry, Faculty of Medicine, Khon Kaen University, Khon Kaen, Thailand; 6 Department of Parasitology, Faculty of Medicine, Khon Kaen University, Khon Kaen, Thailand; 7 School of Pre Clinic, Institute of Science, Suranaree University of Technology, Nakhon Ratchasima, Thailand; 8 Faculty of Management Technology, Rajamangala University of Technology Isan, Surin Campus, Surin, Thailand; 9 Institute of Biodiversity, Animal Health and Comparative Medicine, University of Glasgow, Graham Kerr Building, Glasgow, G12 8QQ UK; 10 Big Data Institute, Nuffield Department of Medicine, University of Oxford, Oxford, OX3 7LF UK; 11 Faculty of Associated Medical Sciences, Khon Kaen University, Khon Kaen, Thailand

**Keywords:** Mini Parasep^®^ SF stool concentrator kit, Kato-Katz, Formalin-ethyl acetate concentration technique, Diagnostic performance, Opisthorchiasis, Helminthiasis, *Opisthorchis viverrini*

## Abstract

**Background:**

Control and elimination of the liver fluke (*Opisthorchis viverrini*) is a primary preventive strategy against cholangiocarcinoma in Southeast Asia. A sensitive parasitological diagnostic method is required to facilitate a surveillance and control program. In this study, we evaluated the performance of Mini Parasep^®^ SF stool concentrator kit (stool kit) compared with Kato-Katz (KK) and the quantitative formalin-ethyl acetate concentration technique (FECT) for detection of *O. viverrini* and co-endemic parasitic infections.

**Methods:**

A cross-sectional survey for parasitic infection in residents aged > 15 years in a community in Kalasin province, Northeast Thailand, was conducted in 2018. Fecal samples were collected and screened by KK method, and a subset of samples was further examined by the stool kit and FECT methods. The results were analyzed for prevalence of parasitic infections in addition to the diagnostic performance of the methods for qualitative and quantitative detection of helminthiases.

**Results:**

The initial survey of parasitic infection determined by the KK method (*n* = 567) showed the prevalence of *O. viverrini* was 32.63%, followed by *Taenia* 2.65%, echinostomes 1.76%, hookworms 1.41%, *Trichuris trichiura* 0.53% and *Strongyloides stercoralis* 0.53%. Within a subset of samples tested with multiple diagnostics (*n* = 150), the detection rates of *O. viverrini* by the stool kit, FECT and KK methods were 27.3%, 30.7% and 28.7%, respectively. The diagnostic sensitivity for opisthorchiasis was similar for FECT (75.5%), KK(66.0%) and the stool kit (67.3%). For other parasitic infections, FECT and stool kit methods performed better than KK, particularly in detecting minute intestinal flukes (MIF), *S. stercoralis* and coinfections. When measuring the intensity of *O. viverrini* infection (fecal egg counts), the stool kit results showed a significant positive correlation with KK and FECT (*P* < 0.05).

**Conclusions:**

As the stool kit is simple to use and shows a comparable performance to FECT, it may serve as an alternative method of fecal examination for screening of helminthiasis including opisthorchiasis.

**Graphical abstract:**

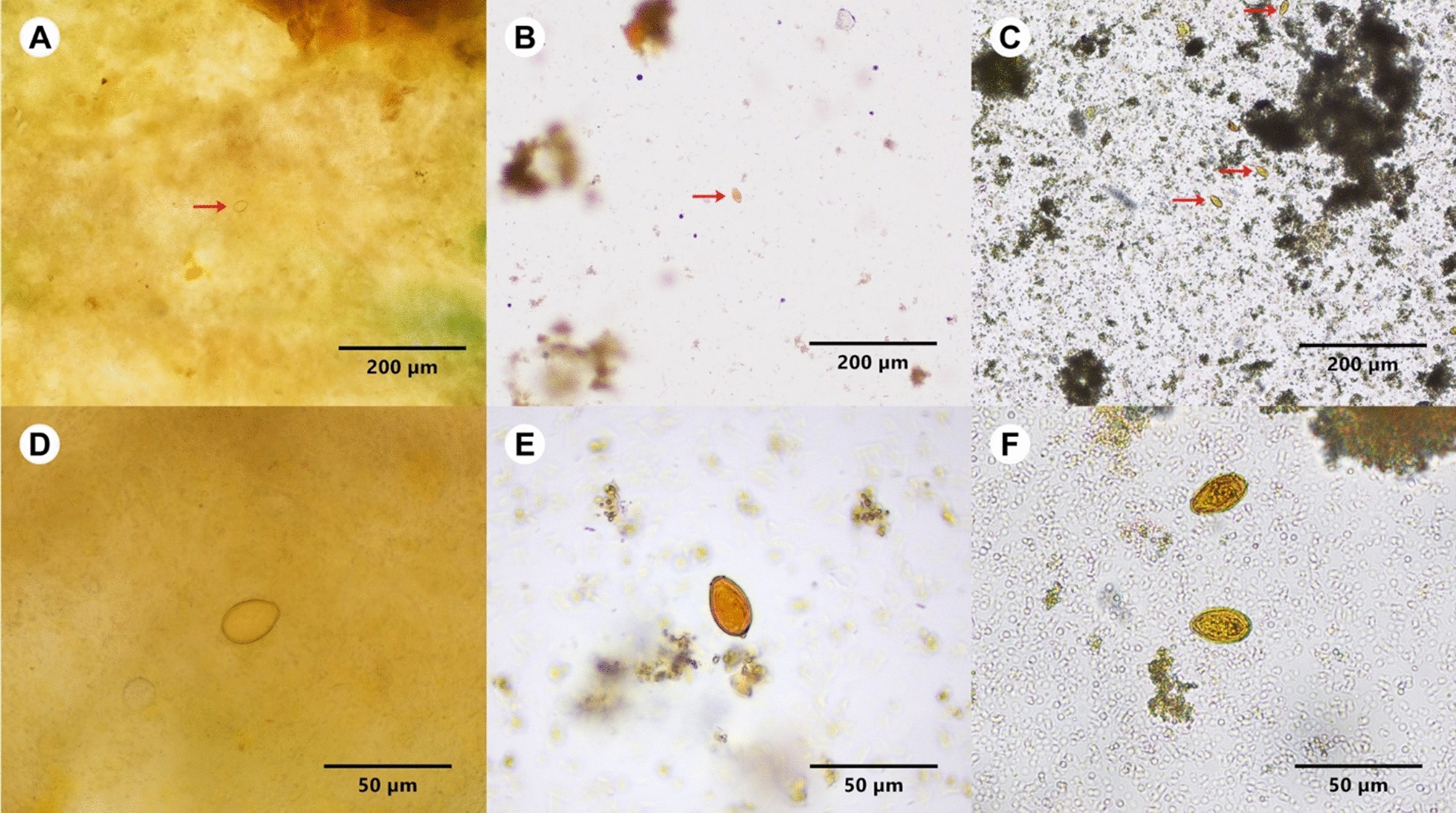

**Supplementary Information:**

The online version contains supplementary material available at 10.1186/s13071-022-05338-z.

## Background

*Opisthorchis viverrini* infection is a major public health problem in the Mekong River Basin region of Southeast Asia, especially in Thailand, the Lao People’s Democratic Republic (Lao PDR), Cambodia and Vietnam [1, 2]. The clinical sequela of chronic opisthorchiasis is hepatobiliary pathology, particularly intrahepatic cholangiocarcinoma (CCA) [3, 4]. Consequently, *O. viverrini* has been classified as a Group I biological carcinogen by the International Agency on Research in Cancer [5]. As *O. viverrini* infection has a fundamental role in the induction of CCA and the resulting fatalities, a comprehensive strategy for prevention, control and elimination of *O. viverrini* is a prerequisite step toward reduction of the incidence of CCA [6].

After decades of chemotherapeutic control and improvement of public health efforts to control the parasitic diseases in Thailand, the epidemiology of *O. viverrini* infection has changed dramatically [7]. Currently, *O. viverrini* infections are maily light (eggs/gram feces < 50) and spread across extensive geographical regions, especially in North and Northeastern Thailand, where control programs have been implemented for decades [2, 8, 9]. Screening of individuals with opisthorchiasis for surveillance and control in endemic areas has traditionally relied on conventional parasitological methods, i.e., Kato-thick smear [10], formalin-ethyl acetate concentration technique (FECT), Kato-Katz (KK) method and simple smear method [11]. These methods are known to have several drawbacks, including limited analytical sensitivity, i.e., low-intensity infections can go undetected and may require repeated fecal examination over several days. In addition, the KK method has been shown to have limited analytical specificity, with *O. viverrini* eggs often confused with the eggs from minute intestinal flukes (MIF) such as *Phaneropsolus bonnei* and *Prosthodendrium molenkampi*, and accurate distinction requires experienced microscopists [12–15]. A previous study suggested that FECT is superior to the KK method for screening of *O. viverrini*, but FECT is logistically complicated, i.e., samples require centrifugation, and has practical disadvantages compared with the KK method [16]. To ease the complications of performing FECT, stool concentrator kits such as the Mini Parasep^®^ Kit have been developed to minimize specimen handling within a disposable enclosed system [17]. In addition, after specimen preparation, the samples can be kept for later examination, which eases time demands when screening large numbers of people. Initial studies using the stool kit have suggested that the method has comparable or better diagnostic performance than the direct simple smear; however, it was reported to be less sensitive than FECT and KK methods for diagnosis of *O. viverrini* [16, 18]. We previously compared the stool kit against FECT and found that the stool kit method was less sensitive in the detection of *O. viverrini* but had a comparable performance for other co-endemic helminth infections [19]. As the results from these preliminary studies have been conflicting and based on small sample sizes, more data are needed to further evaluate the performance of the stool kit for detecting *O. viverrini* as well as other helminth infections in different transmission settings.

The objective of this study was to (i) perform a cross-sectional survey of parasitic helminth infection in an endemic area of opisthorchiasis in Northeast Thailand and (ii) evaluate the diagnostic performances of the fecal examination methods including the Mini Parasep^®^ SF stool concentrator kit, KK and FECT for the diagnosis of *O. viverrini* and other parasitic infections. Relationships of quantitative data, i.e., fecal egg counts of *O. viverrini* estimated by each method, were also examined. The results from this study are informative on the current status of parasitic infection in the region and provide additional results on the analytical performance of the commercial stool kit and its feasibility for applications in larger field studies for surveillance and control of opisthorchiasis across endemic countries in Southeast Asia.

## Methods

### Study participants and sample collection

**Fig. 1:**
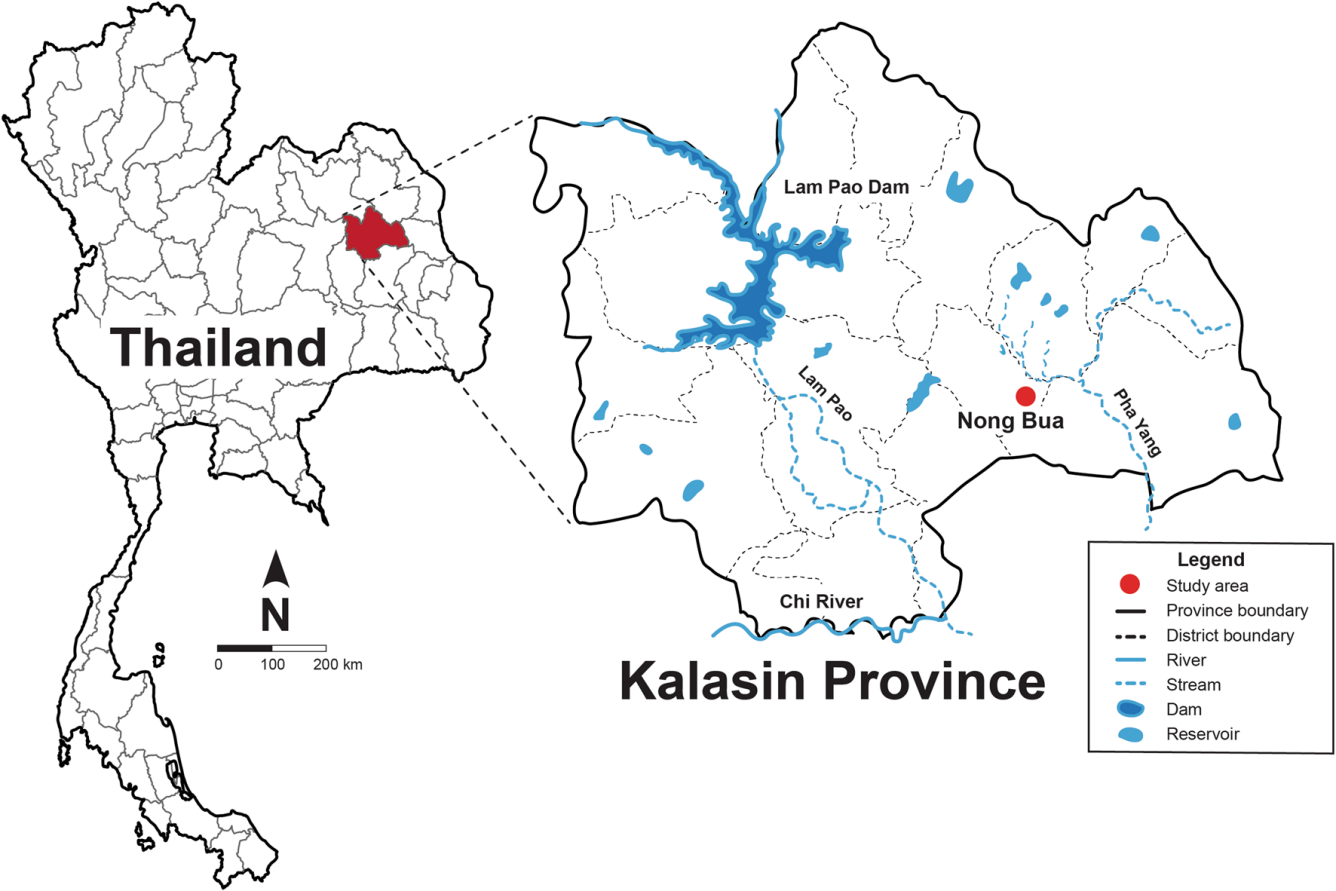
Map of the study area showing an overview of an endemic area for opisthorchiasis in Na Mon district, Kalasin province, northeast Thailand

A cross-sectional survey was carried out from March to July 2018 in an endemic area for infection with *O. viverrini* and other helminth parasites in Na Mon district, Kalasin province, Northeast Thailand (Fig. 1). Male and female residents aged 15 years or older were recruited. The study population mostly comprised farmers working in paddy fields. After written informed consent was obtained, the participants were registered for demographic information. Clean plastic containers were labeled with identifying numbers and were distributed to the villagers to collect the fecal samples. Approximately 10 g of fecal sample was obtained from each participant, and the samples were kept in a chilled insulated box and transported from the study site to the laboratory at the Khon Kaen University. Each fecal sample was thoroughly homogenized using a clean wooden stick to ensure a uniform distribution of parasite eggs and processed for KK (*n* = 567). A subset of 150 samples with a sufficient quantity of stool was additionally processed for FECT (2 g) and Mini Parasep^®^ SF stool concentrator kit (0.5 g).

### Methods and procedures for fecal examination

#### Kato-Katz method

After thorough homogenization of the samples, approximately 1 g of feces was pressed through a mesh screen to remove large debris. Then, a portion of the sieved sample was transferred to the standard template with a hole of 9 mm diameter × 1 mm thickness holding on a slide [20]. After filling the hole, the template was removed, and approximately 41.7 mg of the remaining sample was covered with a piece of cellophane pre-soaked for 24 h in the 3% malachite green-glycerol solution. The slide was allowed to clear for 30 min before examination under a microscope. The number of eggs was counted and recorded for each helminth species separately and multiplied by 23 to calculate the EPG [21, 22].

#### Formalin-ethyl acetate concentration technique (FECT)

Fecal examination using a modified FECT diagnostic method was conducted as reported previously [23, 24]. Briefly, fecal samples were homogenized, and 2 g of fresh feces was weighed and diluted in 7 ml of 10% formalin solution and strained through two layers of gauze into the 15-ml centrifuge tube. The strained suspension was centrifuged at 500*g* for 5 min, and the supernatant was poured out. Thereafter, 7 ml of 0.85% saline was added to the tube and then vigorously mixed with 3 ml of ethyl acetate for 30–60 s to aid in the extraction of fat from the feces. The fecal suspension was centrifuged at 500*g* for 5 min, and the supernatant was discarded and the resulted sediment fixed with 1 ml of 10% formalin. The final fecal suspension was examined with two drops of 40 µl per sample by the same microscopist using a compound microscope at 100× and 400× magnifications with the results combined and multiplied by the number of drops in the suspension and divided by the mass of stool in grams to calculate the number of eggs per gram of feces (EPG). Morphological differentiation between eggs of *O. viverrini* and minute intestinal fluke was done as previously described by Kaewkes [13].

#### Stool kit method

The procedure for the stool kit (Mini Parasep^®^ SF fecal parasite concentrator kit) method (Diasys Europe, Berkshire, UK) was performed according to the manufacturer’s instructions. In short, a fecal sample (~ 0.5 g) was introduced into a tube containing 3.3 ml of 10% formalin solution, and one drop of Triton x-100 then was added. The sample was vortexed to emulsify the mixture. The tube was inverted and centrifuged at 500*g* for 2 min, and then the top layers of the supernatant were decanted. The pellet was resuspended in 1 ml of 10% formalin solution, and two drops, mixed with Lugol’s iodine solution were examined under a light microscope (100×–400× magnification). Enumeration of the eggs per gram of feces (EPG) was similar to the procedure in modified FECT (see above). Discrimination between eggs of *O. viverrini* and minute intestinal fluke (MIF), i.e., *Phaneropsolus bonnei* and *Prostodendrium molenkapi*, was based on morphological characteristics as previously described [13, 25].

### Sample size calculation

An established framework was used to calculate the sample size needed for a comparison between diagnostic tests [26]. Considering that the expected prevalence of *O. viverrini* is 30%, the expected sensitivity of the diagnostic tests is 0.75 [16], the specificity is 0.95, and the precision (difference between methods) is ± 0.15; the required minimum sample size is therefore 107 to detect a significant difference between diagnostic methods with 95% confidence [27].

### Statistical analysis

Data were recorded in case report forms, entered into an Excel worksheet (Microsoft) and analyzed using SPSS 26 (IBM, Chicago, IL, USA). Data from individuals with matched fecal samples by the three diagnostic tests were considered for analysis on diagnostic performance. Positive fecal examination results referred to the presence of at least one parasite egg or larva in the fecal specimen examined by any one of three methods: stool kit, FECT or KK. The gold standard for diagnostic performance (100% specificity and sensitivity) was defined as a combined result from the other two methods, i.e., for FECT the gold standard was defined as the combined results from KK and stool kit method.

Diagnostic accuracy in terms of sensitivity, specificity, positive predictive values (PPV), negative predictive values (NPV) and 95% confidence intervals (95% CI) for total parasites and *O. viverrini* separately were determined using MedCalc (Med Calc Software, Ostend, Belgium). The agreement in the status of *O. viverrini* and other helminth infections between any two methods was evaluated using Cohen’s kappa coefficient. Cohen’s kappa values < 0 were interpreted as indicating no agreement between methods: 0–0.20 as slight, 0.21–0.40 as fair, 0.41–0.60 as moderate, 0.61–0.80 as substantial and 0.81–1 as almost perfect agreement on the status of *O. viverrini* and other helminth infection between different methods [28]. McNemar’s chi-squared test was used to compare the prevalence of *O. viverrini* and other parasitic infections between diagnostic methods.

For analysis on the quantitative relationships between methods, Kendall’s tau-b correlation test was used to determine the correlation of intensity (EPG) between two different methods. A statistically significant level was set at a *P*-value of < 0.05.

## Results

### Prevalence of parasite infection

**Fig. 2:**
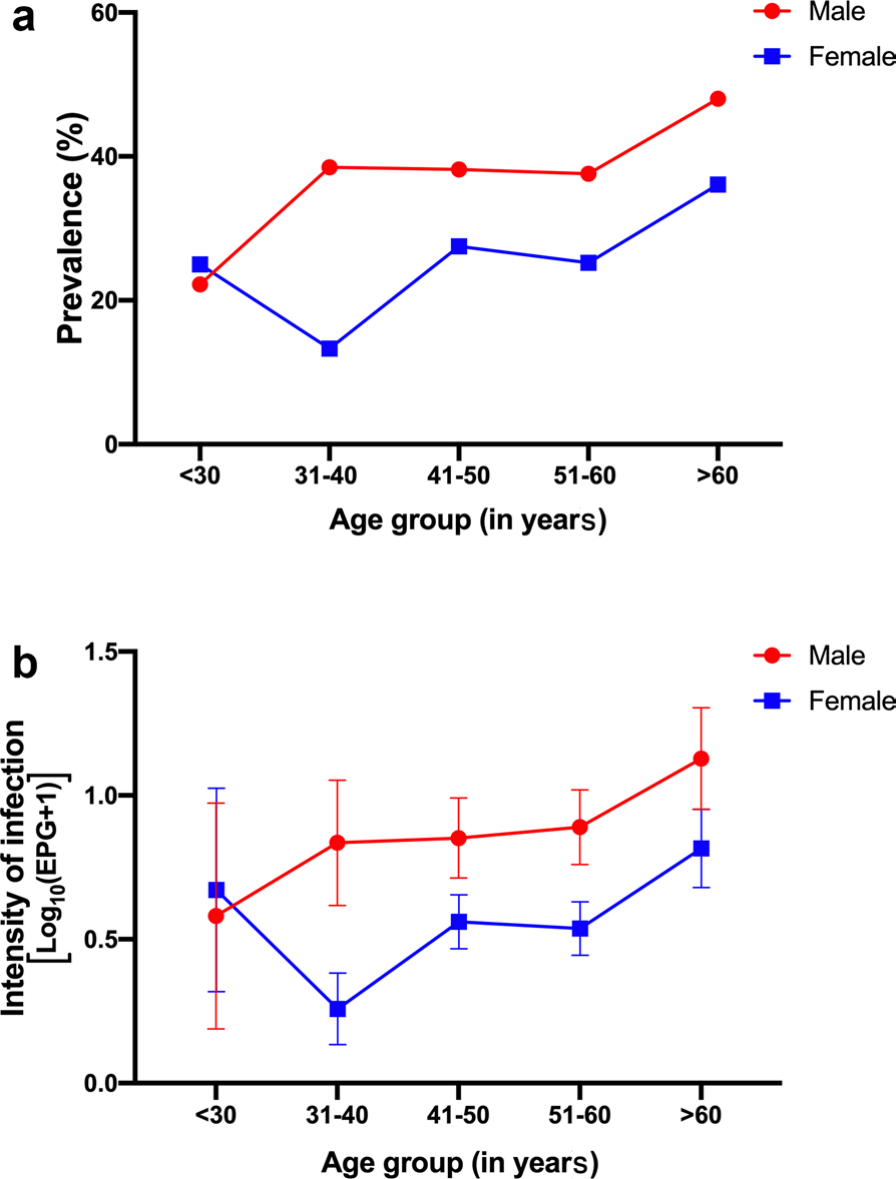
Age-sex prevalence (**a**) and age-sex intensity (**b**) profiles of *O. viverrini* determined by Kato-Katz method (*n* = 567)

From the survey of parasitic infection determined by KK (*n* = 567), 185 individuals were positive for *O. viverrini* infection giving a prevalence of 32.63%. In addition, the observed prevalence for other parasitic helminths was 2.65% for *Taenia* spp., 1.76% for echinostomes, 1.41% for hookworms, 0.53% for *Trichuris trichiura* and 0.53% for *Strongyloides stercoralis*. As shown in Fig. 2, based on the KK method alone, the prevalence and intensity of *O. viverrini* by age group increased gradually and peaked at an age > 60 years. The prevalence of *O. viverrini* in males (95 of 241, 39.42%) was higher than those in females (90 of 328, 27.44%) (*χ*^2^ = 9.53, *df* = 1, *P* < 0.01) (Fig. 2a), and the intensity of *O. viverrini* infection in males was higher than in females (Mann-Whitney *U*-test, *U* = 33,466, *Z* = − 3.571, *P* < 0.001) (Fig. 2b).

**Fig. 3:**
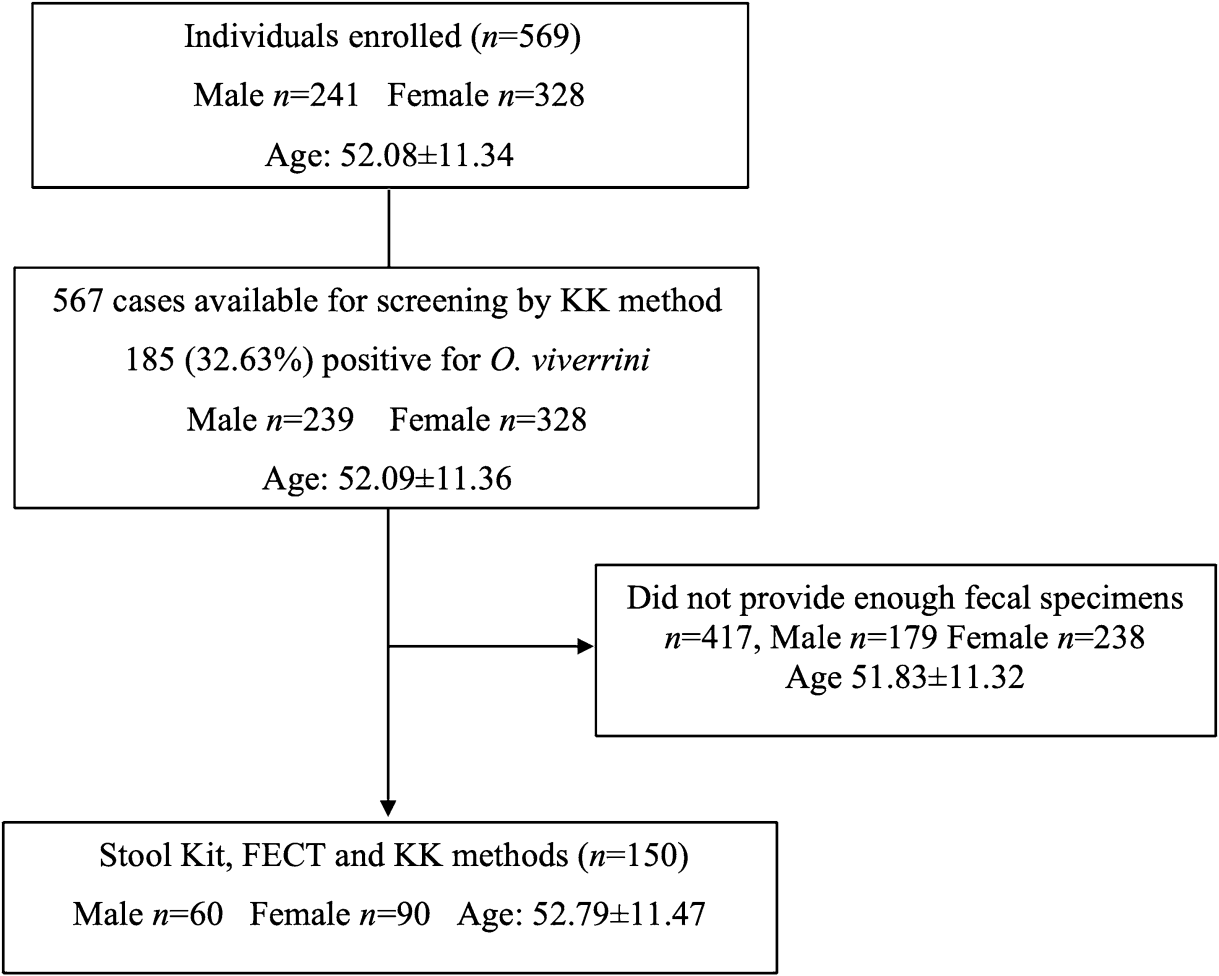
Flow diagram of study participants and sample collection. Data shown for age are mean ± SD, where *n* is the sample size

One hundred fifty participants providing an adequate mass of stool for the three diagnostics were recruited for further study (Fig. 3). The age range of the participants was 17 to 80 years old; 60 were men (40.0%) and 90 women (60.0%). The remaining 417 individuals were excluded from the final analysis as they submitted an insufficient amount of stool. There was no significant difference for sex (LRT = 0.536, *P* = 0.464) and age categories (LRT = 4.174, *P* = 0.525) between the recruited and excluded participants.

### Qualitative comparisons between diagnostic methods for *O. viverrini*

Fecal examinations for *O. viverrini* by the stool kit, FECT and KK methods gave comparable positive detection rates of 30.7% for FECT, 28.7% for KK and 27.3% for stool kit with no statistically significant difference between methods (Table 1 and Additional file 1: Fig. S1). The sensitivity of the stool kit, FECT and KK methods was 67.3%, 75.5% and 66.0%, respectively, for detection of *O. viverrini*. The FECT diagnostic had the highest NPV (88.5%) followed by the stool kit (84.4%) and KK (83.2%). The stool kit had the highest PPV (85.4%) for detecting *O. viverrini* eggs, whereas the FECT had the least PPV (80.4%) (Table 2). There were substantial agreements between the stool kit and FECT (kappa = 0.693), stool kit and KK (kappa = 0.669) and FECT and KK (kappa = 0.665) (Table 3).

**Table 1:** Parasite detection rates determined by Mini Parasep SF kit (stool kit), formalin-ethyl acetate concentration technique (FECT) and Kato-Katz (KK) method and statistical comparisons between methods (*n* = 150)

Parasites	No. positive (%)			*P*-values from McNemar’s test		
	Stool kit	FECT	KK	Stool kit vs FECT	Stool kit vs KK	FECT vs KK
*Opisthorchis viverrini*	41 (27.3)	46 (30.7)	43 (28.7)	0.359	0.824	0.664
Other parasites						
Minute intestinal fluke	17 (11.3)	22 (14.7)	0 (0.0)			
* Strongyloides stercoralis*	16 (10.7)	12 (8.0)	0 (0.0)			
* Echinostome* eggs	3 (2.0)	2 (1.3)	3 (2.0)			
* Taenia* sp.	2 (1.3)	3 (2.0)	3 (2.0)			
Hookworm eggs	3 (2.0)	0 (0.0)	0 (0.0)			
* Ascaris lumbricoides*	0 (0.0)	1 (0.7)	0 (0.0)			
* Trichuris trichiura*	0 (0.0)	1 (0.7)	0 (0.0)			
Combined other parasites	41 (27.3)	41 (27.3)	6 (4.0)	1.000	< 0.001*	< 0.001*
Total parasites	60 (40.0)	61 (40.7)	46 (30.7)	1.000	< 0.05*	< 0.05*

Data shown are the number of positive cases (percentage) and *P*-values

^*^Statistically significant

**Table 2:** Diagnostic accuracy of the stool kit, formalin-ethyl acetate concentration technique (FECT) and Kato-Katz (KK) in the diagnosis of *Opisthorchis viverrini* and overall parasitic infection in fecal samples using a combined result from the other two methods as a reference diagnosis

Method	Sensitivity^a^	Specificity^a^	PPV^a^	NPV^a^
*Opisthorchis viverrini*				
Stool kit	67.3 (52.9–79.8)	93.9 (87.2–97.7)	85.4 (72.4–92.8)	84.4 (78.5–88.9)
FECT	75.5 (61.1–86.7)	91.1 (83.8–95.8)	80.4 (68.4–88.7)	88.5 (82.4–92.6)
KK	66.0 (51.7–78.5)	91.8 (84.4–96.4)	81.4 (68.7–89.7)	83.2 (77.2–87.9)
Overall parasites				
Stool kit	72.7 (60.4–83.0)	85.7 (76.4–92.4)	80.0 (69.9–87.3)	80.0 (72.8–85.7)
FECT	74.2 (62.0–84.2)	85.7 (76.4–92.4)	80.3 (70.4–87.5)	80.9 (73.6–86.6)
KK	56.8 (44.7–68.2)	94.7 (87.1–98.5)	91.3 (79.9–96.5)	69.2 (63.3–74.6)

^a^% (95% CI), positive predictive value (PPV), negative predictive value (NPV)

**Table 3:** Diagnostic agreements among stool kit, FECT and KK method for diagnosis of overall parasite and *Opisthorchis viverrini* infection

Variable	Kappa	95% confidence interval		*P*-values
		Lower	Upper	
*Opisthorchis viverrini*				
Stool kit vs FECT	0.693	0.565	0.820	< 0.001*
Stool kit vs KK	0.669	0.536	0.803	< 0.001*
FECT vs KK	0.665	0.533	0.796	< 0.001*
Overall parasite				
Stool kit vs FECT	0.626	0.499	0.753	< 0.001*
Stool kit vs KK	0.567	0.432	0.701	< 0.001*
FECT vs KK	0.612	0.483	0.741	< 0.001*

Data shown are Cohen’s kappa statistics and *P* value from *Z*-scores

^*^Statistically significant

### Comparisons between quantitative diagnostic for *O. viverrini* (eggs per gram of stool)

**Fig. 4:**
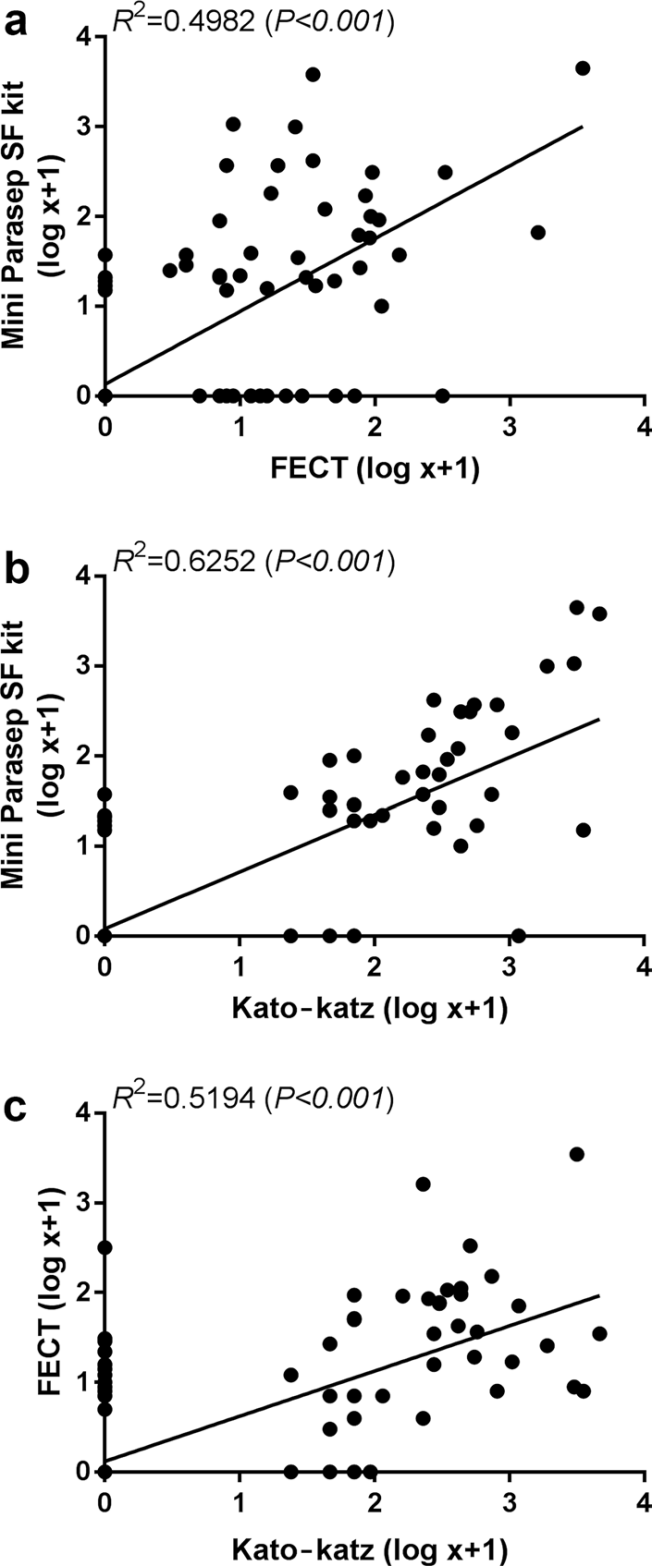
Relationship between log eggs per gram of feces of *O. viverrini* measured by Mini Parasep^®^ SF kit (stool kit) and formalin-ethyl acetate concentration technique (FECT) (**a**), stool kit and Kato-Katz (KK) (**b**) and FECT and KK methods (**c**). The dots shown are observed data and a solid line represented a linear regression equation. *R*^2^ is the correlation coefficient and *P*-value

The intensity of *O. viverrini* infection expressed as log-transformed EPG of feces between stool kit and FECT was positively correlated in a linear regression, with a slope of 0.811 and *r*^2^ = 0.498, *P* < 0.001. We also observed a significant positive correlation of *O. viverrini* eggs between stool kit and KK methods, with a correlation coefficient of 0.625 (slope = 0.635, *r*^2^ = 0.625, *P* < 0.001) (Fig. 4). Eleven cases which tested negative by stool kit had had a range of 23–1173 EPG enumerated by the KK method. Nine cases which tested negative by the KK method had a range of 14–36 EPG reported by the stool kit.

### Differentiating *O. viverrini* and minute intestinal fluke infections

Examining the detection of *O. viverrini* and MIF, which have morphologically similar eggs, revealed that the KK method misclassified three cases of mono-infection with MIF as *O. viverrini.* Mono-infection with MIF was detected in five samples by the stool kit method and six samples by FECT. Mixed *O. viverrini* and MIF infections (24 of 150, 16.0%) were detected by both stool kit and FECT; 12 mixed infections were reported by stool kit, and 16 mixed infections were detected by FECT.

### Comparisons between diagnostic methods for helminth species

Regarding the diagnosis of multiple parasite species infections, 78 participants (52.0%) were found to be infected with at least one helminth (Table 4). FECT and stool kit detected more positive cases of other parasites such as *Strongyloides stercoralis* and MIF than the KK method (McNemar’s test, *P* < 0.001). As shown in Table 2, the stool kit and FECT had significantly higher sensitivity (72.7% and 74.2%) for detecting all parasite species compared with the KK method (McNemar’s test, *P* < 0.05). The test for agreement for all parasite detection revealed a substantial agreement between stool kit and FECT (kappa value = 0.626), FECT and KK (kappa value = 0.612) and moderate agreement between stool kit and KK (kappa value = 0.567) (Table 3).

**Table 4:** Effect of multiple infections on the performance of Mini Parasep SF kit (stool kit), formalin-ethyl acetate concentration technique (FECT) and Kato-Katz (KK) method

Infection category	No. positive (%)			*P*-values from McNemar’s test		
	Stool kit	FECT	KK	Stool kit vs FECT	Stool kit vs KK	FECT vs KK
Single infection	41 (27.3)	40 (26.7)	43 (28.7)	1.000	0.880	0.749
Double infection	16 (10.7)	16 (10.7)	3 (2.0)	1.000	0.002*	0.002*
Multiple infection	3 (2.0)	5 (3.3)	0 (0.0)	0.688	0.250	0.063

^*^Statistically significant

## Discussion

We compared the performance of three methods, formalin-ether concentration technique (FECT), Kato-Katz (KK) and the stool kit method, to diagnose infection with *O. viverrini* and other helminths in a community in Northeast Thailand. For qualitative and quantitative diagnosis of *O. viverrini*, the three methods showed a comparable performance as evaluated by sensitivity, specificity and negative predictive value. The kappa coefficient also indicated “substantial” agreement among the three tests. For diagnosis of other parasitic helminth infections, FECT and stool kit methods showed significantly higher sensitivity than the KK method as greater numbers of co-endemic parasitic infections were detected, particularly MIF and *S. stercoralis*.

The observation that the stool kit performs equally well as FECT for population screening of *O. viverrini*, in addition to other helminth infections, suggests that this diagnostic method is appropriate for surveillance of helminthiasis, including opisthorchiasis. The stool kit may be effective for monitoring the efficacy of mass treatment by anthelmintics; however, this requires examination in future field studies. A known limitation of the KK method is that it does not distinguish MIF from *O. viverrini* [12, 29, 30]. In this study, FECT and stool kit discovered MIF in 11.3–14.7% of tested individuals (*n* = 150) compared to zero cases detected by KK. Although adult worm expulsion and/or molecular confirmation is required for definite identification of MIF [13, 14, 30, 31], previous studies in this part of Northeast Thailand have shown that *O. viverrini* is the predominant infection and the prevalence of MIF was low [23, 32, 33]. By contrast in the central region of Lao PDR MIF, specifically *Haplorchis* spp., were more abundant than *O. viverrini* [34]. Additionally, a study in Northern Thailand, which used molecular diagnostic, found the occurrence of MIF to be 3.8-fold higher than *O. viverrini* [35].

For diagnosis of all helminth infections combined, the FECT and stool kit methods showed higher sensitivity than the KK method. The differences in sensitivity were caused by the KK method failing to detect any *S. stercoralis* larva in the fecal specimens and the inability of the KK method to differentiate between MIF and *O. viverrini.* In case of echinostomes and *Taenia* spp. eggs, which are relatively large, the KK method had a comparable detection rate to FECT and stool kit; this finding has also been shown in previous studies [15, 19, 36, 37]. The agreements between methods for diagnosing all helminths were shown by Cohen’s kappa to range from “moderate” to “substantial.”

As epidemiological surveys of parasitic helminths in Southeast Asia, including Thailand, frequently report coinfections with multiple species [12, 31, 32, 38], diagnostic methods which can reliably classify helminth eggs from different species are preferred. There are several factors which can explain the discrepancy between the different tests. The first is related to the greater amount of fecal sample used by the FECT (2 g) and stool kit (0.5 g) method compared with the KK method (40 mg) [19]. Second, the processing steps in FECT and stool kit which filter out fecal debris as well as fat from fecal specimens contribute to improved egg isolation, hence increasing the chances of detecting parasite eggs or larvae. Also, the protocol for fecal examination by FECT and stool kit was two replicates while it was one replicate for KK. If more replicates had been examined in the KK method, the diagnostic performance might have been improved [37, 39, 40].

Quantitative analysis of fecal egg counts of *O. viverrini* indicated that the diagnostic performances of FECT, stool kit and KK method were similar regardless of the intensity of infection. Given the current trend of widespread low intensity, stool kit and KK method were equally sensitive to discovering *O. viverrini* infected cases. In Korea and China, by contrast, several studies have reported that the KK method was more suitable for *C. sinensis* than the formalin-ether technique and direct smear [37, 39–42]. The KK method has been used for screening and control programs in Thailand for decades [7]. However, in current epidemiological settings in Thailand where light infection of *O. viverrini* is more common, the prevalence of *O. viverrini*, as well as coexisting infections measured by the KK method, is liable to underreporting [42]. While FECT requires specific laboratory equipment and is impractical in resource-poor settings, the stool kit was designed to be an all-in-one tube with a fixative and built-in filtration apparatus and thus removes fecal debris similarly to the procedure for FECT. The stool kit method allows for storage of the sample after preparation, and it can be kept for examination later on; this presents advantages over Kato-Katz, which must be read by technicians within 30 min of slide preparation. Our finding that the stool kit performed equally well to FECT, and better than the KK method, in a parasite survey in Northeast Thailand suggests that it may become the method of choice for future field applications [16, 43].

This study was confined to a single locality; therefore, further applications of the stool kit in multiple localities with varying transmission levels are required for a more comprehensive evaluation of its performance. The second limitation was the examination of a single stool sample taken on 1 day, whereas it is known that replicate examinations may improve diagnostic performance for any fecal diagnostic. Third, the separation of *O. viverrini* and MIF was based on morphological criteria; however, an independent technique such as molecular or adult worm expulsion is required for definite identification and taxonomic confirmation [11]. Evaluation of the cost-effectiveness of the diagnostic methods, which is an essential prerequisite for mass screening, has not been addressed and deserves a separate, detailed study.

## Conclusions

The results from this study demonstrate that FECT, stool kit and KK methods had comparable diagnostic sensitivity for *O. viverrini*. The KK method had lower sensitivity for detection of overall parasitic infections compared with FECT and stool kit. FECT and stool kit performed better for detection of MIF, *S. stercoralis* and multiple infections than KK method. In terms of quantitative diagnosis, fecal *O. viverrini* egg counts measured by the stool kit had a significant positive correlation with those by KK as well as by FECT. Overall, the stool kit has the potential for being a useful tool for screening of *O. viverrini* and coexisting parasitic infections. The utility of the stool kit to assess outcomes following anthelmintic treatment and the cost effectiveness compared to other diagnostics remain to be investigated.

## Supplementary Information

**Additional file 1: Fig S1.** Photomicrographs of *Opisthorchis viverrini* eggs under a light microscopy in different methods. Arrows indicate *O. viverrini* eggs. Panel a and d: Kato-Katz method; panel b and e: FECT; panel c and f: stool kit method. Original magnification ×100, Scale 200 µm or magnification ×400, Scale 50 µm as displayed in the figures.

## Data Availability

The datasets generated and/or analyzed during the current study are available from the corresponding author on reasonable request.

## Abbreviations

CCA: Cholangiocarcinoma
KK: Kato-Katz
Stool kit: Mini Parasep^®^ SF kit
FECT: Formalin-ethyl acetate concentration technique
MIF: Minute intestinal fluke
EPG: Egg per gram feces
MOPH: Ministry of Public Health
PPV: Positive predictive values
NPV: Negative predictive values
95% CI: 95% Confidence intervals
