# Supplementary figures and images for: Performance of Mini Parasep® SF stool concentrator kit, Kato-Katz, and formalin-ethyl acetate concentration methods for diagnosis of opisthorchiasis in Northeast Thailand
Source: Parasit Vectors. 2022 Jun 27;15:234. doi: 10.1186/s13071-022-05338-z (PMC9235228; doi:10.1186/s13071-022-05338-z)

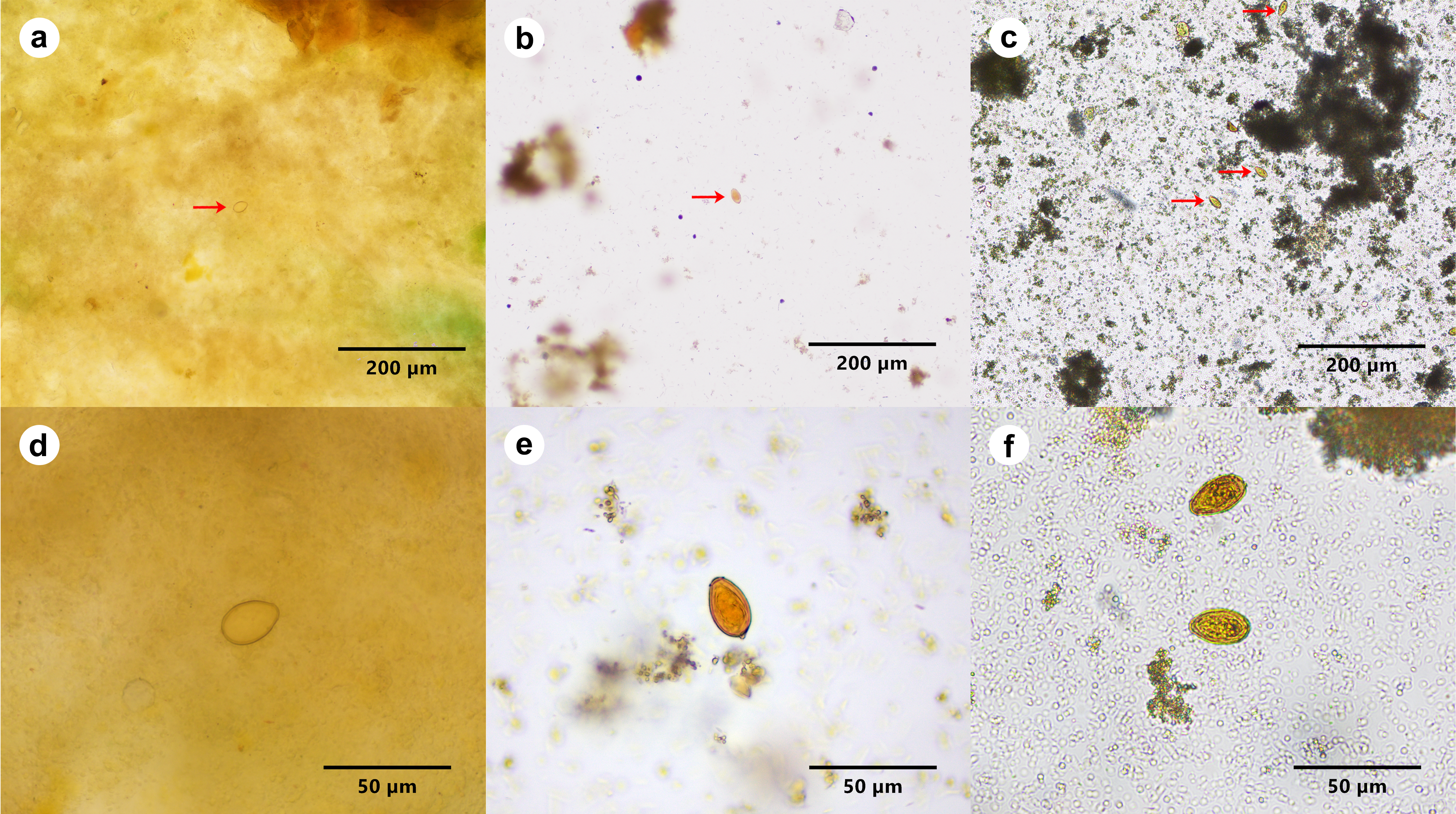

Supplement: Supplementary file 1 — Additional file 1: Fig S1. Photomicrographs of Opisthorchis viverrini eggs under a light microscopy in different methods. Arrows indicate O. viverrini eggs. Panel a and d: Kato-Katz method; panel b and e: FECT; panel c and f: stool kit method. Original magnification ×100, Scale 200 µm or magnification ×400, Scale 50 µm as displayed in the figures. [file 13071_2022_5338_MOESM1_ESM.tif]
